# MicroRNA-26a targets MAPK6 to inhibit smooth muscle cell proliferation and vein graft neointimal hyperplasia

**DOI:** 10.1038/srep46602

**Published:** 2017-04-21

**Authors:** Juanjuan Tan, Liguo Yang, Cuicui Liu, Zhiqiang Yan

**Affiliations:** 1 School of Life Sciences and Biotechnology, Shanghai Jiao Tong University, Shanghai, 200240, P. R. China; 2 Department of Cardiology, Shanghai Jiao Tong University afliated Sixth People’s Hospital South Campus, Shanghai, 201400, P. R. China; 3 Central laboratory, Shanghai Jiao Tong University afliated Sixth People’s Hospital South Campus, Shanghai, 201400, P. R. China

## Abstract

Neointima formation is the major reason for vein graft failure. However, the underlying mechanism is unclear. The aim of this study was to determine the role of miR-26a in the development of neointimal hyperplasia of autogenous vein grafts. Using autologous jugular vein grafts in the rat carotid artery as a model, we found that miR-26a was significantly downregulated in grafted veins as well as proliferating vascular smooth muscle cells (VSMCs) stimulated with platelet-derived growth factor-BB (PDGF-BB). Overexpression of miR-26a reduced the proliferation and migration of VSMCs. Further analysis revealed that the effects of miR-26a in VSMCs were mediated by targeting MAPK6 at the mRNA and protein levels. Luciferase assays showed that miR-26a repressed wild type (WT) MAPK6-3′-UTR-luciferase activity but not mutant MAPK6-3′-UTR-luciferease reporter. MAPK6 deficiency reduced proliferation and migration; in contrast, overexpression of MAPK6 enhanced the proliferation and migration of VSMCs. This study confirmed that neointimal hyperplasia in vein grafts was reduced *in vivo* by up-regulated miR-26a expression. In conclusion, our results showed that miR-26a is an important regulator of VSMC functions and neointimal hyperplasia, suggesting that miR-26a may be a potential therapeutic target for autologous vein graft diseases.

Although autologous vein grafting remains an effective and durable treatment for many patients with atherosclerotic occlusive diseases of the coronary or peripheral circulations^1^,^2^,^3^, vein graft failure as a result of neointimal formation and superimposed atherosclerosis is found in up to 50% of cases in the past decade^4^. A major cause of vein graft failure is intimal hyperplasia, which predominantly results from proliferation and migration of vascular smooth muscle cells (VSMCs) and the deposition of extracellular matrix^5^. VSMCs are one of the principal components in the vasculature and play important roles in maintaining vessel tone and blood pressure. In contrast to most mature cells, VSMCs are remarkably plastic and can dedifferentiate in response to environmental cues^6^,^7^, such as vessel injuries, growth factors and cytokines, including platelet-derived growth factor-BB (PDGF-BB), fibroblast growth factor, insulin-like growth factor-1, tumor necrosis factor-alpha (TNF-a), and interleukin-1^8^,^9^. Specifically, PDGF-BB increases VSMC proliferation and subsequent migration into the neointima layer after artery injury^10^. However, the molecular mechanism by which VSMCs proliferate and migrate after vascular injury is not completely defined.

MicroRNAs are a recently discovered class of endogenous non-coding RNAs that play key roles in the regulation of gene expression. Mature microRNAs are short, single-stranded RNA molecules of approximately 22 nucleotides in length. Acting at the post-transcriptional level, these molecules can fine-tune the expression of as many as 30% of all mammalian protein-encoding genes by binding to the specific 3′ untranslated regions of messenger RNA (mRNA) transcripts and inducing their degradation or translational repression^11^,^12^. The biogenesis of miRNAs is under tight temporal and spatial control, and their dysregulation is associated with many human diseases, particularly cancer^13^. MicroRNAs are highly expressed in the cardiovascular system, and they have been implicated in the development of cardiovascular diseases, including atherosclerosis^14^,^15^,^16^. MiR-26a was shown to play a dual role in promoting or inhibiting tumorigenesis^17^,^18^. For example, miR-26a promotes tumor angiogenesis in glioma, while it suppresses tumor-associated angiogenesis in hepatocellular carcinoma^17^,^19^. Interestingly, ectopic expression of miR-26a significantly induced endothelial cell cycle arrest and inhibited migration, sprouting angiogenesis, and network tube formation in matrigel *in vitro* and *in vivo*^20^. MiR-26a up-regulation in ASMCs^Des−/−^ induced hypertrophy via suppression of the anti-hypertrophic protein GSK-3β^21^. However, it remains unclear whether miR-26a regulates the function of rat jugular vein smooth muscle cells, which are associated with neointimal formation resulting from vein graft failure.

In this study, we report that miR-26a is an important regulator of VSMC functions and neointimal hyperplasia by targeting MAPK6 at the gene level. Our study provides a better understanding of neointimal formation associated with vein graft failure.

## Results

### Down-regulation of miR-26a expression in the proliferation of rat jugular vein VSMCs

**Figure 1:**
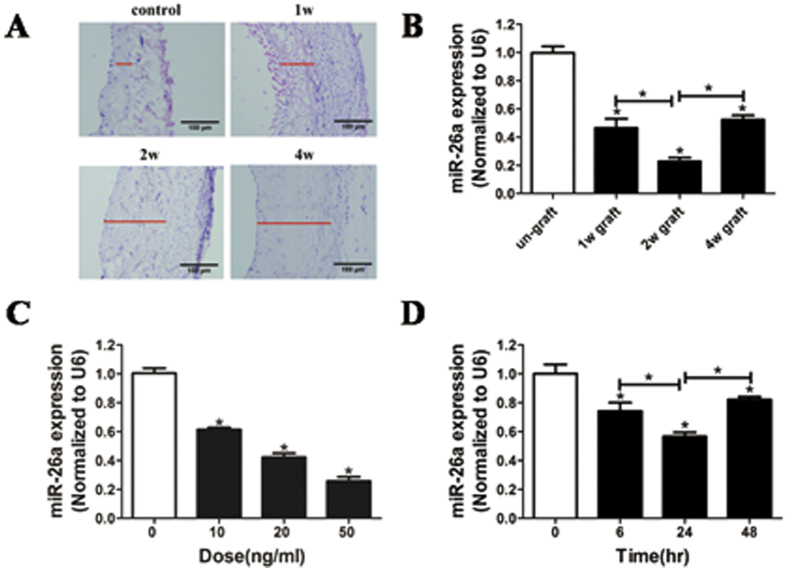
Down-regulation of miR-26a expression is linked to proliferation of rat jugular vein VSMCs. (**A**) Representative HE staining of control (Ctrl) veins and vein grafts at 1, 2, and 4 weeks after operation. (**B**) Down-regulation of miR-26a expression in grafted-veins at 1, 2, and 4 weeks after autologous jugular vein graft. *P < 0.05 represents statistical significance compared to the un-grafted group. (**C**) Decrease in miR-26a expression induced by platelet-derived growth factor-BB (PDGF-BB; 20 ng/ml) in a dose-dependent manner in rat jugular vein SMCs as determined by quantitative reverse-transcription polymerase chain reaction (qRT-PCR) (n = 4). *P < 0.05 represents statistical significance compared to the treatment with PDGF-BB for 0 h. (**D**) PDGF-BB caused a time-dependent decrease in miR-26a expression in rat jugular vein VSMCs 24 h after PDGF-BB treatment, as determined by qRT-PCR (n = 4). *P < 0.05 represents statistical significance compared to treatment with 0 ng/ml PDGF-BB.

To investigate whether miR-26a is a critical regulator for VSMC proliferation in the neointima, we used a well-established model of rat autologous jugular vein grafts. Neointimal lesions of the rat jugular vein were induced (Fig. 1A), and the expression of miR-26a in jugular veins was significantly down-regulated (Fig. 1B) at week 1, 2, and 4 after autologous jugular vein graft, suggesting that miR-26a may be a critical regulator for VSMCs in the vascular wall. Furthermore, we found that miR-26a expression in VSMCs was significantly down-regulated after treatment with PDGF-BB in a dose-dependent and time-dependent manner (Fig. 1C and D). These results indicated that miR-26a may be a critical regulator of VSMC proliferation.

### Inhibition of VSMC proliferation by miR-26a

**Figure 2:**
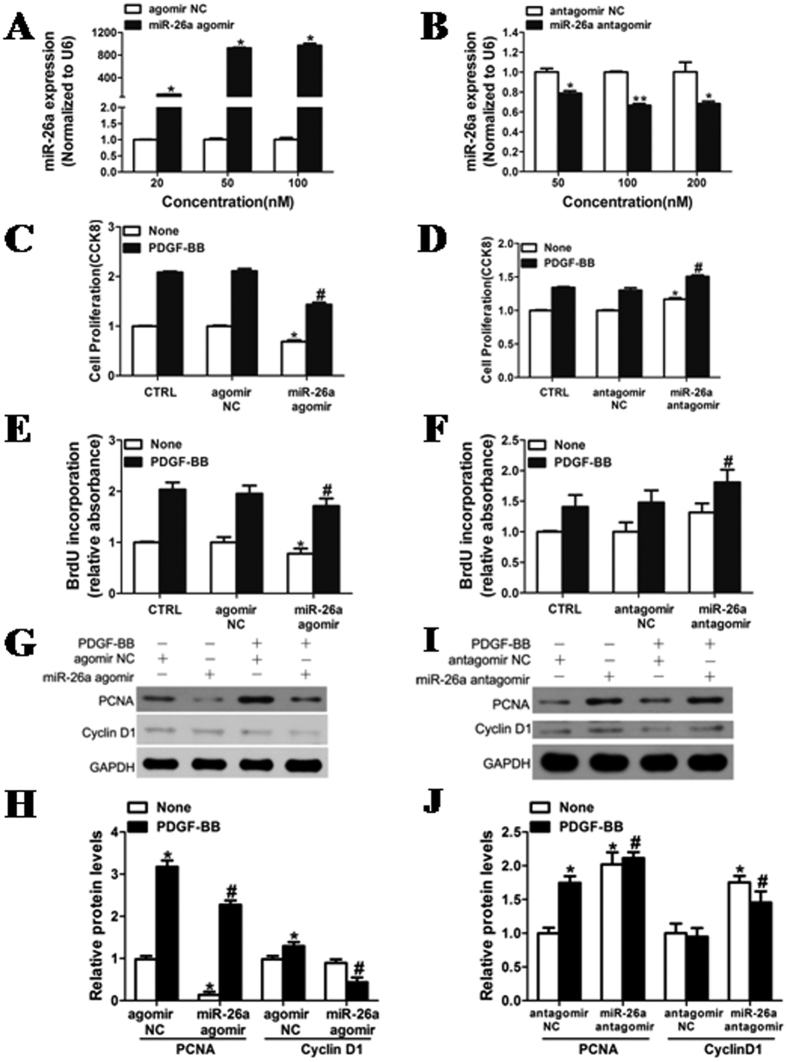
Inhibition of VSMC proliferation by miR-26a. (**A,B**) Rat jugular vein VSMCs were transfected with agomir NC, miR-26a agomir, antagomir NC or miR-26a antagomir at different concentrations for 48 h, followed by analysis with quantitative reverse-transcription polymerase chain reaction (qRT-PCR) for miR-26a level. (**C**,**E)** Rat jugular vein VSMCs transfected with miR-26a agomir (50 nM) or agomir NC (50 nM) were stimulated with or without PDGF-BB (20 ng/ml) for 24 h. The proliferation of VSMCs transfected with miR-26a agomir was significantly inhibited after stimulation with PDGF-BB in rat jugular veins, as determined by CCK8 proliferation assays and BrdU incorporation. (**D**,**F**) Rat jugular vein VSMCs transfected with miR-26a antagomir (100 nM) or antagomir NC (100 nM) were stimulated with or without PDGF-BB (5 ng/ml) for 24 h. The proliferation of rat jugular vein VSMCs transfected with miR-26a antagomir was enhanced by PDGF-BB as determined by CCK8 proliferation assays and BrdU incorporation. (**G**) Rat jugular vein VSMCs transfected with miR-26a agomir (50 nM) or agomir NC (50 nM) were stimulated with PDGF-BB (20 ng/ml) for 24 h followed by analysis of PCNA and CyclinD1 protein levels with western blotting. The original images for (**G**) can be seen in Supplementary Fig. 1. (**H**) Densitometric analysis of PCNA and CyclinD1 protein levels as determined by western blotting. (**I**) Rat jugular vein VSMCs transfected with miR-26a antagomir (100 nM) or antagomir NC (100 nM) were stimulated with or without PDGF-BB (5 ng/ml) for 24 h followed by analysis of PCNA and CyclinD1 protein levels with western blotting. The original images for (**I**) can be seen in Supplementary Fig. 4. (**J**) Densitometric analysis of PCNA and CyclinD1 protein levels as determined by western blotting. *P < 0.05 represents statistical significance compared with the negative control without PDGF-BB, ^#^*P* < 0.05 represents statistical significance compared with the negative control with PDGF-BB (n = 4).

To confirm the role of miR-26a in inhibiting VSMC proliferation, VSMCs were transfected with miR-26a agomir, miR-26a antagomir or negative controls. Figure 2A and B show that the expression of miR-26a was increased in a dose-dependent manner in the VSMCs transfected with miR-26a agomir, whereas endogenous levels of miR-26a were substantially reduced in the VSMCs transfected with miR-26a antagomir. Accordingly, the cell proliferation induced by PDGF-BB was significantly inhibited in VSMCs transfected with miR-26a agomir (Fig. 2C and E). In contrast, cell proliferation was promoted in VSMCs transfected with miR-26a antagomir in response to low-dose PDGF-BB (Fig. 2D and 2F). The effect of miR-26a on VSMC proliferation was further confirmed by the significantly attenuated expression of PCNA and Cyclin D1, regulators of VSMC proliferation (Fig. 2G and H), in VSMCs transfected with miR-26a agomir after stimulation with PDGF-BB. Together, these results indicate that miR-26a plays an important role in regulating the proliferation of VSMCs from the jugular veins of rats.

### Inhibition of VSMC *in vitro* wound repair by miR-26a

**Figure 3:**
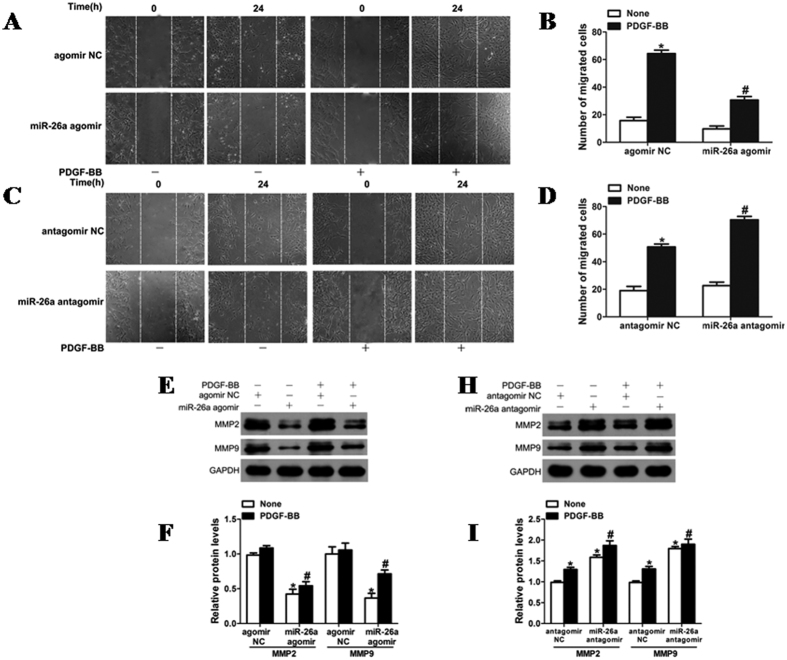
Role of miR-26a in the migration of VSMCs. (**A**) VSMCs transfected with miR-26a agomir oligos were starved, and cell migration was measured after stimulation with PDGF-BB (20 ng/ml) for 24 h by scratch-wound assays. (**B**) Migrated cells were quantitated, and the results are displayed as the mean ± SD. The experiment was repeated three times. *P < 0.05 represents statistical significance compared with VSMCs with agomir NC but without PDGF-BB; ^#^*P* < 0.05 represents statistical significance compared with VSMCs with agomir NC treated with PDGF-BB. (**C**) VSMCs transfected with miR-26a antagomir (100 nM) or antagomir NC (100 nM) were used for scratch assays, thus creating a gap in the cell monolayer followed by stimulation with or without PDGF-BB (5 ng/ml) for 24 h. The scratch-healing status was monitored. Each scratch was photographed, and the width was measured using Image-Pro Plus software (Media Cybernetics, Rockville, MD). Results were quantitated by calculating the mean width of the wound. (**D**) Migrated cells were quantitated, and the results are displayed as the mean ± SD. The experiment was repeated three times. *P < 0.05 represents statistical significance compared with VSMCs with antagomir NC but without PDGF-BB; ^#^*P* < 0.05 represents statistical significance compared with VSMCs with antagomir NC treated with PDGF-BB. (**E**) Rat jugular vein VSMCs transfected with miR-26a agomir (50 nM) or agomir NC (50 nM) were stimulated with PDGF-BB (20 ng/ml) for 24 h followed by analysis of MMP-2 and MMP-9 protein levels with western blotting. The original images for (**E**) can be seen in Supplementary Fig. 5. (**F**) Densitometric analysis of MMP-2 and MMP-9 protein levels as determined by western blotting. (**H**) Rat jugular vein VSMCs transfected with miR-26a antagomir (100 nM) or antagomir NC (100 nM) were stimulated with or without PDGF-BB (5 ng/ml) for 24 h followed by analysis of MMP-2 and MMP-9 protein levels with western blotting. The original images for (**H**) can be seen in Supplementary Fig. 4. (**I**) Densitometric analysis of MMP-2 and MMP-9 protein levels as determined by western blotting. ^#^*P* < 0.05 represents statistical significance compared with the control.

Furthermore, miR-26a was associated with VSMC migration. Overexpression of miR-26a via transfection with agomir delayed the wound closure in a scratch model of VSMC monolayers under both basal and PDGF-BB-stimulated conditions (Fig. 3A and B). Both basal and PDGF-induced VSMC migration was augmented in VSMCs transfected with miR-26a antagomir (Fig. 3Cand D). In addition, MMP-2 and MMP-9, which are implicated in VSMC migration^22^, were significantly inhibited in VSMCs overexpressing miR-26a (Fig. 3E). These results indicate that miR-26a is an inhibitor of VSMC *in vitro* wound repair.

### MiR-26a functions in VSMCs by directly targeting MAPK6

**Figure 4:**
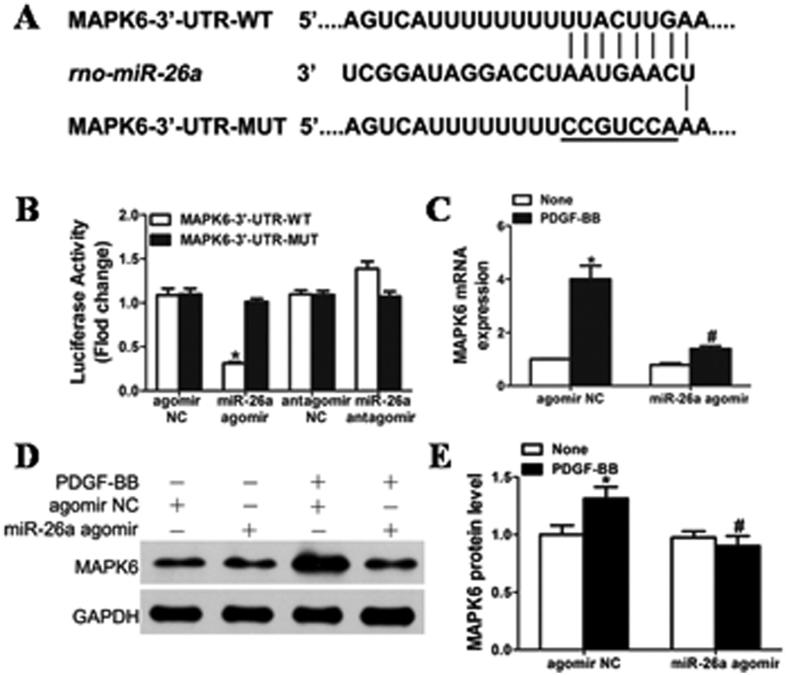
The role of miR-26a in VSMCs by directly targeting MAPK6. (**A**) Schematic of the miR-26a putative binding site in rat MAPK6 3′-UTR and alignment of wild-type and mutated MAPK6 3′- UTR binding sites of miR-26a. The seven mutated nucleotides are underlined. (**B**) miR-26a agomir (50 nM) or antagomir (100 nM) was co-transfected with the luciferase reporter carrying WT-MAPK6 3′-UTR or mutated MAPK6 3′-UTR. Forty-eight hours after transfection, Renilla luciferase activities were measured (n = 5). *P < 0.05 represents statistical significance compared with MAPK6-3′-UTR-WT transfected with agomir NC. (**C**) VSMCs transfected with miR-26a agomir or agomir NC were stimulated with or without PDGF-BB (20 ng/ml) for 24 h, and MAPK6 mRNA was measured by qRT–PCR in rat jugular vein VSMCs. *P < 0.05 represents statistical significance compared with agomir NC without PDGF-BB; ^#^*P* < 0.05 represents statistical significance compared with agomir NC with PDGF-BB. (**D**) Effects of overexpression of miR-26a on the expression of MAPK6 in VSMCs treated with PDGF-BB (20 ng/ml). The original images for (**D**) can be seen in Supplementary Fig. 1. (**E**) Densitometric analysis for the levels of MAPK6 protein by western blot. *P < 0.05 represents statistical significance compared with agomir NC without PDGF-BB, ^#^*P* < 0.05 represents statistical significance compared with agomir NC with PDGF-BB.

**Figure 5:**
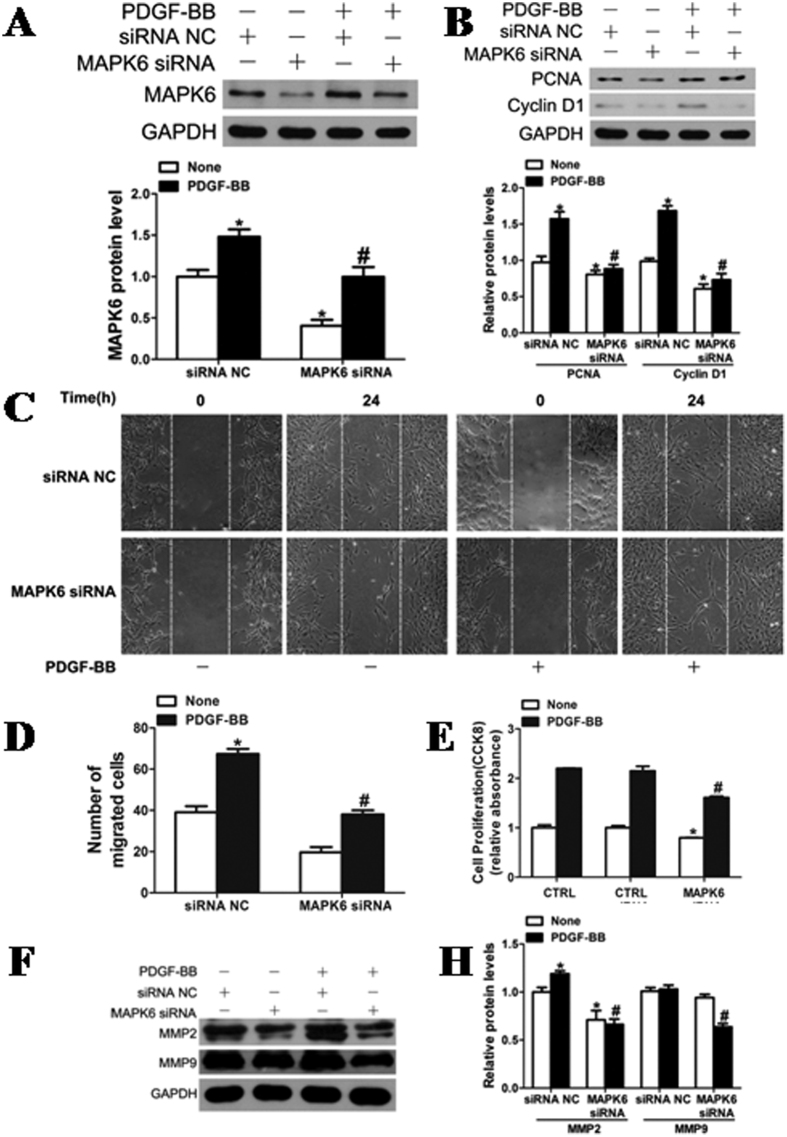
Role of MAPK6 in VSMC proliferation and migration. (**A**) MAPK6 expression was reduced by silencing MAPK6 with specific small interfering RNA (MAPK6 siRNA; 100 nM). *P < 0.05 represents statistical significance compared with the control siRNA without PDGF-BB, ^#^*P* < 0.05 represents statistical significance compared with the control siRNA with PDGF-BB. (**B**) The expression of PCNA and Cyclin D1 was reduced after silencing MAPK6. *P < 0.05 represents statistical significance compared with control siRNA without PDGF-BB, ^#^*P* < 0.05 represents statistical significance compared with control siRNA with PDGF-BB. The original images for (**A** and **B**) can be seen in Supplementary Fig. 2. (**C**) VSMCs transfected with MAPK6 siRNA (100 nM) were assessed for cell migration after stimulation with PDGF-BB (20 ng/ml) for 24 h by scratch-wound assays. (**D**) Quantitation of migrated cells. The data are shown as the mean ± SD of the number of migrated cells from 3 independent experiments. *P < 0.05 represents statistical significance compared with the control siRNA without PDGF-BB, ^#^*P* < 0.05 represents statistical significance compared with the control siRNA with PDGF-BB. (**E**) MAPK6 deficiency attenuated PDGF-BB (20 ng/ml)-induced proliferation of VSMCs as determined by CCK8 assays (n = 4). (**F**) Rat jugular vein VSMCs transfected with MAPK6 siRNA (100 nM) or siRNA NC (100 nM) were stimulated with or without PDGF-BB (20 ng/ml) for 24 h followed by analysis of MMP-2 and MMP-9 protein levels with western blotting. The original images for (**F**) can be seen in Supplementary Fig. 6. (**H**) Densitometric analysis of MMP-2 and MMP-9 protein levels as determined by western blotting. *P < 0.05 represents statistical significance compared with the control siRNA without PDGF-BB, ^#^*P* < 0.05 represents statistical significance compared with the control siRNA with PDGF-BB.

MAPK6 (mitogen-activated protein kinase 6) was a potential miR-26a target based on its mRNA 3′-UTR, which was complementary to miR-26a as determined by TargetScanHuman 6.2 and microrna.org. Figure 4A shows that rat MAPK6 mRNA has a potential miR-26a binding site in its 3′-UTR. To determine whether miR-26a directly binds to the 3′-UTR sequence of rat MAPK6 mRNA and affects its expression, the 3′-UTR sequence of MAPK6 containing the putative binding site of miR-26a was cloned into a pmirGLO Dual-Luciferase miRNA Target Expression vector. The constructed vector was then co-transfected with either the miR-26a agomir or agomir negative control (agomir NC) into HEK293 cells. Figure 4B shows that the luciferase activity was inhibited in the cells transfected with the miR-26a agomir but not in the cells with agomir NC. In contrast, the cells transfected with the miR-26a antagomir showed increased luciferase activity. Furthermore, mutation of the miR-26a binding site in the 3′-UTR of MAPK6 significantly decreased the inhibitory effects of miR-26a on the luciferase activity (Fig. 4B). To further verify that MAPK6 is a functional target gene of miR-26a in rat VSMCs, VSMCs were transfected with either agomir NC or miR-26a agomir (50 nmol/L), and MAPK6 levels were measured by both qRT–PCR (Fig. 4C) and western blot (Fig. 4D and E). Overexpression of miR-26a markedly decreased the expression of MAPK6 in both basal and PDGF-BB-induced conditions. VSMCs transfected with MAPK6 siRNA displayed significantly inhibited basal and PDGF-BB-induced MAPK6 expression (Fig. 5A). Taken together, these results indicated that miR-26a selectively binds to the 3′-UTR of rat MAPK6 mRNA and inhibits its expression in VSMCs.

### Role of MAPK6 in VSMC proliferation and migration

**Figure 6:**
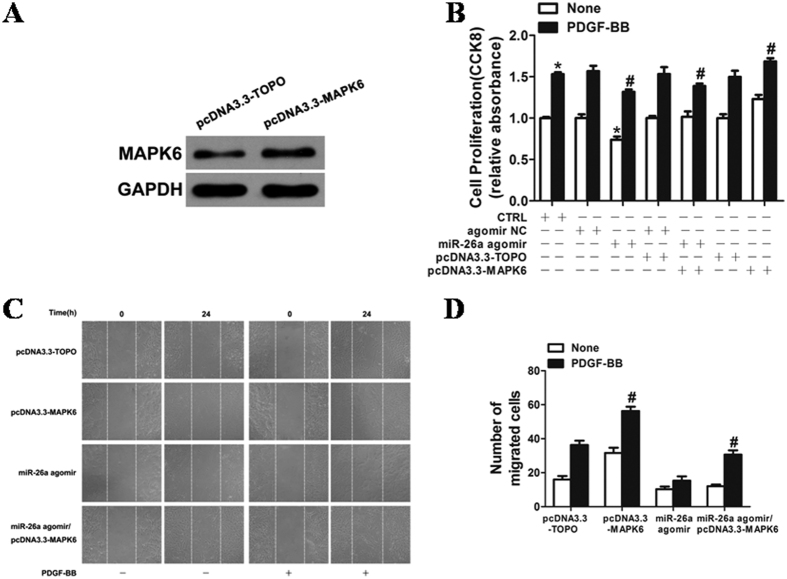
Overexpression of MAPK6 markedly augmented VSMC proliferation and wound closure after stimulation with PDGF-BB. (**A**) VSMCs transfected with pcDNA3.3-MAPK6 significantly increased the expression of MAPK6. MAPK6 expression was determined by western blotting in VSMCs 48 h after transfection. The original images for (**A**) can be seen in Supplementary Fig. 3B–D. Overexpression of MAPK6 markedly augmented the VSMC proliferation and wound closure after stimulation with PDGF-BB. (**B**) VSMCs transfected with either miR-26a agomir or agomir NC (50 nM) were stimulated with or without PDGF-BB (20 ng/ml) for 24 h, and cell proliferation was measured by the CCK8 assay (n = 6). *P < 0.05 represents statistical significance compared with agomir NC without PDGF-BB. ^#^*P* < 0.05 represents statistical significance compared with miR-26a agomir with PDGF-BB. (**C**) VSMCs transfected with oligos were starved, and cell migration was measured after PDGF-BB stimulation for 24 h by the scratch wound assay. (**D**) Migrated cells were quantitated and are shown as the mean ± SD of the number of migrated cells from three independent experiments. *P < 0.05 represents statistical significance compared with the control.

VSMCs with MAPK6 knockdown markedly decreased the expression of PCNA, CyclinD1, MMP-2 and MMP-9 (Fig. 5B and F) and reduced proliferation induced by PDGF-BB (Fig. 5E). Both basal and PDGF-BB-stimulated wound closure of VSMC monolayers were also markedly attenuated in the cells transfected with MAPK6 siRNA (Fig. 5C and D), indicating that MAPK6 is a critical regulator for VSMC proliferation and migration. We found that the VSMCs transfected with pcDNA3.3-MAPK6 significantly increased the expression of MAPK6 (Fig. 6A). Overexpression of MAPK6 markedly augmented the VSMC proliferation and wound closure stimulated by PDGF-BB (Fig. 6B, C and D). The inhibitory effects of miR-26a on PDGF-BB-induced VSMC proliferation and migration were partially reversed by overexpression of MAPK6, suggesting that the inhibitory effect of miR-26a on VSMC proliferation and migration was, at least partly, mediated by directly targeting MAPK6.

### miR-26a inhibits vascular neointimal formation in a rat model of autogenous jugular vein graft

**Figure 7:**
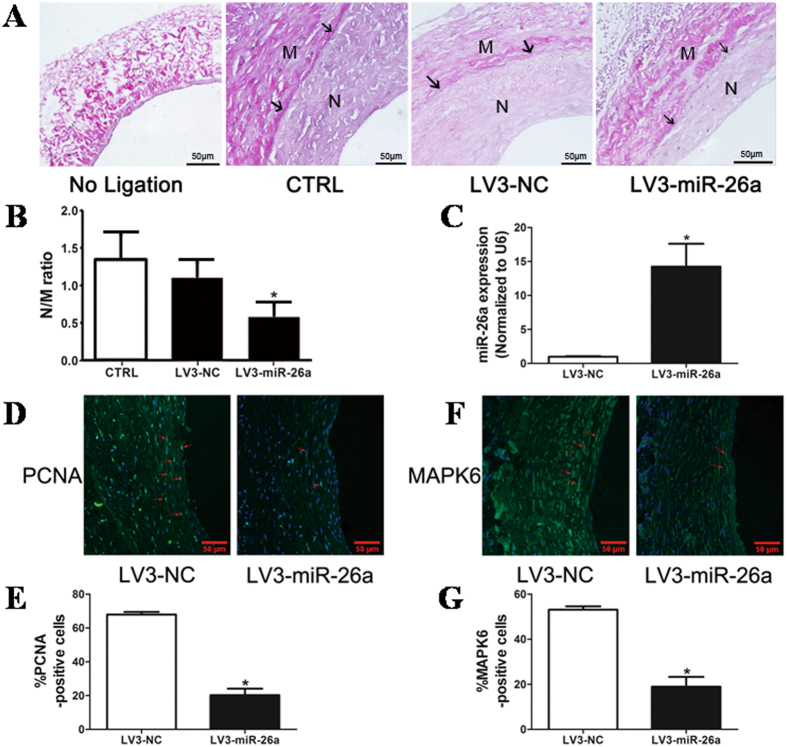
miR-26a attenuates neointimal formation in a rat model of autogenous jugular vein graft. (**A**) LV3-mediated overexpression of miR-26a (LV3-miR-26a) significantly reduced neointimal formation *in vivo*. Representative Elastica van Gieson -stained jugular veins from rats treated with LV3 or LV3-miR-26a 4 weeks after autogenous jugular graft. Black arrows indicate internal elastic membrane. Cross-sectional areas of the intima and media were marked with N and M respectively. (**B**) The effect of miR-26a on vascular neointimal lesion formation in rat jugular veins 4 weeks after autogenous jugular graft as quantitated by neointima/media (N/M) ratio. *P < 0.05 represents statistical significance compared with LV3-NC. (**C**) LV3-miR-26a (10^8^ pfu/ml) or LV3-NC (10^8^ pfu/ml) was suspended in 50 μl Pluronic F127 gel (BASF, 30% wt/vol) and was applied around the grafted-jugular vein. Jugular veins were harvested 4 weeks after graft. Total RNAs were extracted, and the expression of miR-26a was detected by qRT-PCR (n = 4). The expression of miR-26a in rat jugular vein transduced with LV3-NC was undetectable (ND). *P < 0.05 represents statistical significance compared with LV3-NC. (**D**) Representative immunofluorescence staining of PCNA in rat jugular veins 4 weeks after ligation injury. Green indicates PCNA, which represents proliferating cells; blue is the cell nuclear staining by DAPI. (**E**) Quantification of PCNA-positive cells showed that fewer cells were proliferating in the grafted-vein treated with LV3-miR-26a compared with LV3-treated vessels. *P < 0.05 represents statistical significance compared with LV3-NC. (**F**) Representative immunofluorescence staining of MAPK6 in rat jugular vein 4 weeks after ligation injury. Green indicates MAPK6 staining, and blue is nuclear staining by DAPI. (**G**) Quantification of MAPK6-positive cells showed that MAPK6 was downregulated in LV3-miR-26a-treated vessels compared with LV3-treated vessels. *P < 0.05 represents statistical significance compared with LV3-NC.

Interestingly, miR-26a is also involved in neointimal formation in the rat autogenous jugular vein graft model 4 weeks after grafting. The grafted-jugular vein was transduced with either LV3-miR-26a (10^8^ pfu/mL) or LV3-GFP. The expression of miR-26a in the grafted-jugular vein was markedly increased compared with the LV3-GFP-treated group 4 weeks after transduction (Fig. 7C). In contrast, flow enhancement led to a substantial increase in neointimal formation in grafted-jugular veins transduced with either gel alone or the gel with LV3-GFP (Fig. 7A). Transduction of the jugular vein with LV3-miR-26a inhibited the jugular vein graft-induced neointimal formation up to 30%, which was associated with decreases in the intima-to-media ratio (Fig. 7B) in LV3-miR-26a-treated jugular veins. Figure 7D and E show that the ratio of PCNA-positive nuclei to total cells was significantly decreased in LV3-miR-26a-transduced jugular veins compared with the LV3-GFP-treated jugular veins. Moreover, the number of MAPK6-positive cells was also substantially reduced in the LV3-miR-26a-transduced jugular veins (Fig. 7F and G). Taken together, these results suggest that miR-26a inhibits jugular vein graft-induced neointimal formation *in vivo* by suppressing MAPK6-mediated VSMC proliferation.

## Discussion

Although recent studies have examined the role of microRNAs in VSMC biology^23^, the molecular mechanisms by which VSMCs proliferate and migrate and VSMC pathogenesis remain unclear. In this study, miR-26a was identified as a novel modulator involved in rat jugular vein VSMC proliferation and migration because miR-26a was significantly down-regulated in rat proliferative VSMCs and grafted veins, and miR-26a regulated VSMC proliferation and migration *in vitro* and neointimal formation *in vivo*. Further analysis revealed that miR-26a suppresses the expression of MAPK6 and its downstream targets PCNA, Cyclin D1, MMP-2 and MMP-9. Thus, our study identified miR-26a as a novel key regulator that plays an important role in rat jugular vein VSMC biology.

MiR-26, a functional miRNA, has received much attention from researchers in recent years. MiR-26 may play crucial roles in growth and development of normal tissues and the pathogenesis of non-tumor diseases and tumor formation^24^. In tumorigenesis, miRNAs act as oncogenic or tumor-suppressive genes in various tumor types. MiR-26 expression is significantly down-regulated in several types of cancer, such as bladder, breast, hepatocellular carcinoma and oral cancer^25^,^26^,^27^,^28^, and may exhibit tumor-suppressive activity during tumorigenesis in these cancers. For example, miR-26a was down-regulated in breast cancer specimens and cell lines, and it initiated apoptosis through endogenous and exogenous pathways activated by caspase8 and caspase9 as well as through direct binding to the 3′-UTR of MTDH and EZH2. MiR-26 impairs the *in vitro* colony-forming and *in vivo* tumor-loading abilities of MCF7 cells^27^. MiR-26a was significantly decreased in anaplastic carcinomas (ATC) compared to normal thyroid tissue. The overexpression of miR-26 in two human ATC-derived cell lines significantly decreased thyroid carcinogenesis, suggesting a crucial role for miR-26 down-regulation in thyroid carcinogenesis^29^. In contrast, recent studies revealed that the expression of miR-26 was up-regulated in tumors such as glioma^30^,^31^ and cholangiocarcinoma tissues and cell lines^32^. MiR-26 is overexpressed in high-grade glioma and is frequently amplified at the DNA level in a subset of human high-grade gliomas. Overexpression of miR-26a in a murine glioma model revealed that miR-26a effectively repressed endogenous PTEN protein by binding to 3 potential binding sites in the PTEN 3′-UTR in a relevant glioma model system, promoting tumorigenesis. MiR-26 may therefore be oncogenic in glioma. Thus, miR-26 exerts diverse effects on cellular function, either inhibiting or promoting cell proliferation in different cell types^24^. miRNAs are evolutionarily conserved and act at the post-transcriptional level as “fine tuners” and/or “safeguards” to balance dramatic environmentally induced alterations in gene expression and maintain organism homeostasis^33^. Many miRNAs are expressed in a tissue-specific manner, such as cardiac and skeletal-specific miRNAs (miR-1, miR-133, miR-206), which have been shown to regulate muscle development and function^34^,^35^. In addition, many miRNAs are expressed and function in a context-dependent manner. For example, the miR-17-92 cluster is called an “oncomiR” because of its high expression and involvement in cancer^36^. Interestingly, in our study, the expression of miR-26a in jugular veins was significantly down-regulated (Fig. 1B) at week 1, 2, and 4 after autologous jugular vein graft, but down-regulation was most significant at week 2. Similar results were found 24 h after treatment with PDGF-BB in VSMCs (Fig. 1D). These results may reflect the biologically context-dependent expression pattern of miR-26a in autologous-grafted jugular veins and VSMCs treated with PDGF-BB.

In the current study, we showed that miR-26a was down-regulated by PDGF-BB in VSMCs and grafted veins. More importantly, up-regulation of miR-26a inhibited VSMC proliferation and migration and reduced vein graft neointimal hyperplasia, consistent with a previous report showing that ectopic expression of miR-26a inhibited endothelial cell proliferation, migration and angiogenesis^20^. However, the effects of miR-26a on VSMC function were opposite to the findings discovered by Leeper *et al*., who showed that overexpression of miR-26a enhanced human aortic SMC proliferation^37^. MiR-26a was also shown to be abundantly expressed in stretch-induced hypertrophic human airway smooth muscle cells (HASMCs), and enforced expression of miR-26a induced HASMC hypertrophy^38^. These different results may be explained by the different experimental parameters, such as cell lines and histological origin. These results indicate that the diverse effects of miR-26 on VSMC are species-specific and depend on the external stimuli.

MiRNAs regulate approximately 30% of the protein-coding genes in the human genome^39^. Recently, a growing body of target genes, such as TRPC6, LOXL2, CTGF of miR-26a and miR-26b, has been identified^40^,^41^,^42^. In this study, the MAPK6 gene was identified as a direct target of miR-26a in rat jugular vein VSMCs as shown by luciferase reporter assays. Overexpression of miR-26a reduced the MAPK6 expression at both the protein and mRNA levels. MAPK6, also known as extracellular signal-regulated kinase 3 (ERK3), belongs to a group of atypical MAPKs that display a SEG motif in the activation loop (instead of TEY) and have a long C-terminal extension. The regulation, substrate specificity, and physiological functions of atypical MAP kinases are not completely understood^43^. Mitogen-activated protein (MAP) kinases are a superfamily of serine/threonine kinases that play a major role in transducing extracellular chemical and physical signals into intracellular responses^44^. Erk1/2 are a subfamily of the classic MAP kinase family^45^, and the role of Erk1/2 in cell survival is well established. Activation of ERK1/2 has been shown to promote cell survival and inhibit apoptosis in response to a wide range of stimuli, including growth factor withdrawal^46^. In contrast to the well-studied ERK1 and ERK2, much less is known about MAPK6 with regard to its function and the molecular regulation of its signalling (both expression and activation). Only one substrate has been described for MAPK6 thus far, namely, the MAPK-activated protein (MAPKAP) kinase MK5 (also known as PRAK). MK5 binds to ERK3 via a novel MAPK interaction motif^47^. Increased cytoplasmic ERK3 causes the nuclear-cytoplasmic translocation of MK5, the formation of ERK3/MK5 signalling complexes, and the subsequent activation of MK5 by phosphorylation^43^. Recently, MAPK6 was shown to regulate primary human umbilical vein endothelial cell migration, proliferation and angiogenesis. MAPK6 is substantially upregulated by approximately twofold in primary HUVECs treated with both TNF-α and IL-1β cytokines. Knockdown of MAPK6 significantly impaired tube formation of HUVECs^48^. MAPK6 has also been shown to play an important role in cellular differentiation^49^,^50^,^51^. Mice with MAPK6 deficiency display intrauterine growth restriction and neonatal lethality predominantly due to pulmonary immaturity with loss of differentiation of type II pneumocytes^52^. Consistent with these results, we showed here increased expression of MAPK6 in PDGF-BB-treated VSMCs and grafted veins. Knockdown or overexpression of MAPK6 modulated PDGF-BB-induced VSMC proliferation and migration. These results indicate that MAPK6 levels play a key role in maintaining normal physiological conditions for VSMCs.

VSMC proliferation and migration represent a major feature of vein graft neointimal hyperplasia. A number of studies have showed that matrix metalloproteinases and cell cycle-related proteins are implicated in this process^53^. MMP and cell cycle-related protein expression is regulated by growth factors via activation of multiple signalling pathways, such as PI-3K and MAPK^54^,^55^. This study revealed that knockdown of MAPK6 or overexpression of miR-26a inhibited proliferation and migration of VSMC by reducing expression of PCNA, cyclin D1, MMP2 and MMP9.

In summary, our study identified miR-26a as a novel regulator of rat jugular vein VSMCs by targeting, at least partly, the MAPK6 pathway. MiR-26a expression was substantially down-regulated in proliferative rat jugular vein VSMCs, and restoration of its expression markedly inhibited both VSMC proliferation and migration in response to PDGF-BB stimulation. Thus, our study provides novel insight into the molecular mechanisms associated with VSMC proliferation and migration and suggests a potential therapeutic target for preventing and treating human vascular diseases, such as atherosclerosis, restenosis, and vein graft disease.

## Materials and Methods

### Vein graft surgery

Male Sprague-Dawley rats (10–12 weeks old, 300–400 g) were purchased from the Shanghai Laboratory Animal Research Center and housed in a temperature- and light-controlled room (approximately 22 ± 2 °C, 12 h light/dark cycles) with free access to water. The vein grafting surgery was performed as described previously^56^. Briefly, general anesthesia for rats was induced using an isofluorane vaporizer with 5% anesthetic gas and was maintained with 2.5%. After an incision was made in the midline of neck, the left external jugular vein was dissected, and the tributaries were ligatured with 8-0 silk sutures. The segment of jugular vein was harvested and stored in heparin-containing saline (50 IU·mL^−1^). The left common carotid artery was gently mobilized free from surrounding connective tissues, temporarily ligated with two microvascular clamps and then cut in the middle. Each end of the artery was passed through a polymer cuff (made from Braun, inner diameter 0.75 mm, outer diameter 0.94 mm), everted, and fixed with a piece of 8-0 silk suture. The vein segment was then sleeved over the artery cuffs and secured with 8-0 sutures. The arterial clamps were removed, and the blood flow was re-established. The operative field was irrigated thoroughly with saline solution, and the skin incision was closed with 5-0 sutures. The contralateral veins were used as matched controls. The vein grafts were harvested at week 1, 2 and 4 after implantation. Animal care was provided in accordance with the procedures outlined in the “Guidelines on the Conduct of Animal Experiments issued by the Institutional Animal Care and Use Committee of Shanghai Jiao Tong University (Shanghai, China)” and were approved by the Ethics Committees of Shanghai Jiao Tong University.

### Cell culture

Primary rat VSMCs were isolated from jugular veins as described previously with modification^57^. Briefly, VSMCs were cut into pieces and treated with 1 ml (1 mg/ml) collagenase type II (Sigma) and 0.125% trypsin for 1 h and then centrifuged at 1000 rpm for 3 min to harvest the cells after removal of endothelial cells. The cells were grown in Medium 231 with Smooth Muscle Growth Supplement (Gibco), 100 U/ml penicillin and 100 μg/ml streptomycin at 37 °C, 5% CO_2_. The purity of the VSMCs was confirmed by immunofluorescence analysis of smooth muscle α-actin. The cells at passages 3 to 7 were used in all experiments. HEK293 cells were cultured in Dulbecco’s modified Eagle’s medium (DMEM) (Gibco) containing 10% foetal bovine serum (FBS) (Gibco).

### Lentiviral vector-mediated miR-26a mimic delivery into the vein grafts

The lentivirus expressing miR-26a (LV3-miR-26a) was generated using a GM Easy^TM^ Lentiviral Packaging Kit (Genomeditech, China) according to the manufacturer’s instructions. Briefly, the pGMLV vector and GM Easy^TM^ Lentiviral Mix plasmid were co-transfected with the pCDH1-expressing vector containing the miR-26a sequence into HEK293 cells using HG Transgene^TM^ Reagent (Genomeditech, China). The lentiviruses were propagated in HEK293 cells and purified using centrifugation and filtration by 0.45 μm Millipore filter membranes. Titering was assayed using a serial dilution method. For local delivery of the lentiviral vector-mediated miR-26a mimics (LV3-26a) and negative controls (LV3-NC) into grafted veins, we used an established local delivery model with Pluronic F-127 gel (Sigma) as previously described^58^. Vein segments were immersed in 500 μl lentivirus solution (either LV3-miR-26a or LV3-NC, 10^8^ pfu/ml) for 5 min. Then, 250 μl lentivirus solution was preloaded into 250 μl Pluronic F-127 gel (30% wt/vol) at 4 °C and gently painted around the grafted vein segments. Rats were randomly divided into 3 groups: the LV3-miR-26a-treated group, LV3-NC group and the Sham group. The vein grafts were harvested at week 4 after surgery.

### Cell transfection

For miR-26a overexpression, miR-26a agomir (GenePharma, China) was added to the complexes at final concentrations of 50 nM. For miR-26a knockdown, the miR-26a antagomir (GenePharma, China) was added to the complexes at final concentrations of 100 nM. Knockdown of MAPK6 expression was performed using MAPK6 siRNA-smart pool (Dharmacon^TM^), with all negative siRNAs (GenePharma, China) as a control. Overexpression of MAPK6 was performed by inserting full-length rat MAPK6 cDNA into the pcDNA3.3 expression vector. Twelve hours after they were seeded into the 6-well plates, cells were transfected using either RNAi-MATE (GenePharma, China) or PolyJet™ DNA *in vitro* Transfection Reagent (SignaGen Laboratories, China) according to the manufacture’s protocol. Transfection medium was replaced by regular cell culture medium after 6 h of transfection.

### qRT-PCR

Total RNAs were extracted from both tissue and jugular VSMCs with TRIzol Reagent (Invitrogen) according to the manufacturer’s instructions. RNA concentrations were determined with a Nanodrop ND-1000 spectrophotometer (Thermo Fisher Scientific). Quantitative reverse transcriptase (qRT–PCR) was performed in triplicate using a 7500 Real-Time PCR System (Applied Biosystems). qRT–PCR for miRNA was performed using All-in-One^TM^ miRNA first-strand cDNA synthesis kit (GeneCopoeia, China) and SYBR^®^ PremixEx Taq^TM^ (Tli RNaseH Plus) (TaKaRa, China). The primers for qRT-PCR were as follows:

miR-26a

Forward primer: 5′-GCG GCGGTTCAAGTAATCCAGG-3′

Reverse primer: 5′-TGGTGTCGT GGAGTCG-3′

MAPK6

Forward primer: 5′-TAAAGCCATTGACATGTGGG-3′

Reverse primer: 5′-TCGTGCACAACAGGGATAGA-3′

MMP-2

Forward primer: 5′-GACCTTGACC AGAACACCATCG-3′

Reverse primer: 5′-GCTGTATT CCCGACCGTTGAAC-3′

MMP-9

Forward primer: 5′-CCCCACTTACTTTGGAAACGC-3′

Reverse primer: 5′-ACCCACGACGATACAGATGCTG-3′

GAPDH

Forward primer: 5′-ACAAGATGGTGAAGGTCGGTGTGA-3′

Reverse primer: 5′-AGCTTCCCATTCTCAGCCTTGACT-3′

The ratio of miR-26a versus U6 expression and the relative expression levels of MAPK6, MMP-2 and MMP-9 to GAPDH were determined by the 2^−ΔΔCt^ method.

### Western blotting

The western blotting assay was performed as previously described^59^. Briefly, whole cells were lysed with Pierce^®^ RIPA Buffer containing complete protease inhibitor cocktail (Roche). The protein concentration was determined using a BCA protein assay kit (Pierce). Cell lysates were then resolved by SDS-PAGE and transferred to nitrocellulose (Millipore). The membranes were then blocked with 5% nonfat milk in TBS with 0.1% Tween 20 (TBST) followed by incubation with primary antibodies overnight at 4 °C and then incubated with HRP-labelled secondary antibodies. The immunolabelled proteins were detected with a BM Chemiluminescence Western Blotting kit (Roche). Densitometric quantification was performed with a Gel Doc 1000 system and analysed by Quantity One Software (BioRad). The dilutions of primary antibodies were as follows: MAPK6 (Proteintech, #12839-1-AP, 1:500), PCNA (Cell Signaling, #2586, 1:1000), Cyclin D1 (Cell Signaling, #2926, 1:500), and GAPDH (Proteintech, #10494-1-AP, 1:1000).

### Luciferase reporter assay

MAPK6 3′-UTR fragments with wild type (WT) or mutant miR-26a binding sites were cloned by PCR. The primers are as follows:

WT MAPK6 3′-UTR

Forward primer: 5′-AACGAGCTCGCTAGCCTCGAGTCATGAAATGTGTTGTGTCTT-3′

Reverse primer: 5′-CCTGCAGGTCGA CTCTA ACACCATTAAGACTTCTAGGAGC-3′

mutant MAPK6 3′-UTR

Forward primer: 5′-AACGAGCTCGCTAGCCTCGAGTTTCCGTCCAAATCAGAAGGTGT-3′

Reverse primer: 5′-CCTGCAGGTCGACTCTAGACACCATTAAGACTTCTTCCTCG-3′

PCR products were inserted into the pmirGLO vector (Promega) with an In-Fusion HD Cloning Plus system (Clontech). MiRNA target reporter assays were performed in quadruplicate in 96-well plates. Then, 200 ng of each construct or vehicle control was co-transfected with miR-26a agomir or negative control (50 nM) into HEK293 cells using Lipofectamine 2000 according to the recommended protocol. Firefly and Renilla luciferase activities were sequentially measured using the Dual-Glo™ Luciferase Assay system (Promega) with a GloMax microplate luminometer (Promega) 48 h after transfection. Relative luciferase activity was normalized with Renilla luciferase activity.

### Assay for VSMC proliferation

VSMC proliferation was determined by either CCK-8 (Dojindo Molecular Technologies) assays or BrdU incorporation assays (Roche) according to the protocols provided by manufacturers. For the CCK-8 assay, 10 μl of CCK-8 was dispensed into each well of a 96-well culture plate, and the absorbance was measured at 450 nm after 2 h incubation. For the BrdU incorporation assay, BrdU was added to the culture medium for incorporation into cellular DNA. After 4 h of incubation, cells were fixed, and anti-BrdU antibody was added and incubated for 30 min followed by measurement of the absorbance at 450 nm.

### Scratch wound assay

VSMCs were seeded in 6-well plates at a concentration of 2.0 × 10^5^ cells per well. After incubation with starvation medium (1% FBS) for 24 h, a linear scratch wound was gently produced in the center of the cell monolayer using a sterilized 200 μL disposable pipette tip followed by washing with PBS to remove the cellular debris. Cells were stimulated with or without human PDGF-BB at a final concentration of 5 or 20 ng/ml. The wound closure was monitored for an additional 24 h. The closure of scratch wounds was visualized under a microscope (Leica), and the widths of the scratch wounds were measured with Image Pro Plus 6.0 software. Wound closure % indicates the percentage of wound closure, with the initial scratch width as 100%.

### Histomorphometry and immunofluorescence (IF)

The vein grafts were fixed in 4% paraformaldehyde for 24 h, dehydrated, embedded in paraffin, and cut into sections (5 μm). The sections were stained by Haematoxylin & eosin (H&E) staining and Elastica van Gieson staining. Microscopic images from each section were captured with an Olympus microscope (Tokyo). Histomorphometry analysis was performed using ImageJ2x. The values were measured at eight points, and the average value of the four sections was considered to represent the examined grafts. Immunofluorescence staining was performed as follows: after dewaxing in xylene and stepwise rehydration in ethanol, the sections were treated with antigen retrieval for 10 min at 120 °C. The sections treated with normal goat serum for 30 min to reduce nonspecific binding were incubated with anti-MAPK6 rabbit polyclonal antibody (Proteintech, #12839-1-AP, 1:200) or anti-PCNA rabbit polyclonal antibody (Cell Signaling, #2586, 1:500) overnight at 4 °C, followed by fluorescein-conjugated anti-rabbit secondary antibodies (Biotum). Cell nuclei were counterstained with DAPI (Sigma).

### Statistical analysis

Data are expressed as mean ± SD and analysed for statistical significance by unpaired Student’s *t* test or one-way ANOVA using GraphPad Prism 5.0. All *in vitro* experiments were performed at least 3 times. *P* < 0.05 was considered a statistically significant difference in all experiments.

## Additional Information

**How to cite this article**: Tan, J. *et al*. MicroRNA-26a targets MAPK6 to inhibit smooth muscle cell proliferation and vein graft neointimal hyperplasia. *Sci. Rep.* **7**, 46602; doi: 10.1038/srep46602 (2017).

**Publisher's note:** Springer Nature remains neutral with regard to jurisdictional claims in published maps and institutional affiliations.

## Supplementary Material

Supplementary Information
