# Supplementary material for: MicroRNA-26a targets MAPK6 to inhibit smooth muscle cell proliferation and vein graft neointimal hyperplasia
Source: Sci Rep. 2017 Apr 21;7:46602. doi: 10.1038/srep46602 (PMC5399463; doi:10.1038/srep46602)
Supplement: Supplementary Information [file srep46602-s1.doc]

**MicroRNA-26a targets MAPK6 to inhibit smooth muscle cell proliferation and vein graft neo****intimal hyperplasia**

**Juanjuan Tan^1^, Liguo Yang^2^, Cuicui Liu^2^ and Zhiqiang Yan^2^***

1. School of Life Sciences and Biotechnology, Shanghai Jiao Tong University

2. Shanghai Jiao Tong University Affiliated Sixth People’s Hospital South Campus

*Correspondence to: Zhiqiang Yan, Shanghai Jiao Tong University Affiliated sixth People’s Hospital South Campus, No.6600 Nanfen Road,P.R.China,200240, *E-mail:zqyanshanghai@ gmail.com*


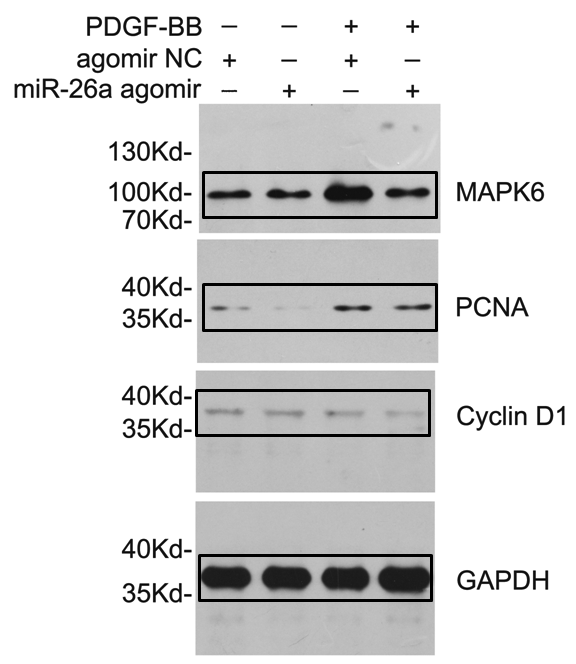


Supplementary Figure 1. Relative MAPK6,PCNA and Cyclin D1 protein expressions analyzed with western blots in agomir NC and miR-26a agomir with or without PDGF-BB lysates. GAPDH is the loading control.The boxed regions showed the content appearing in the figures in the main text.


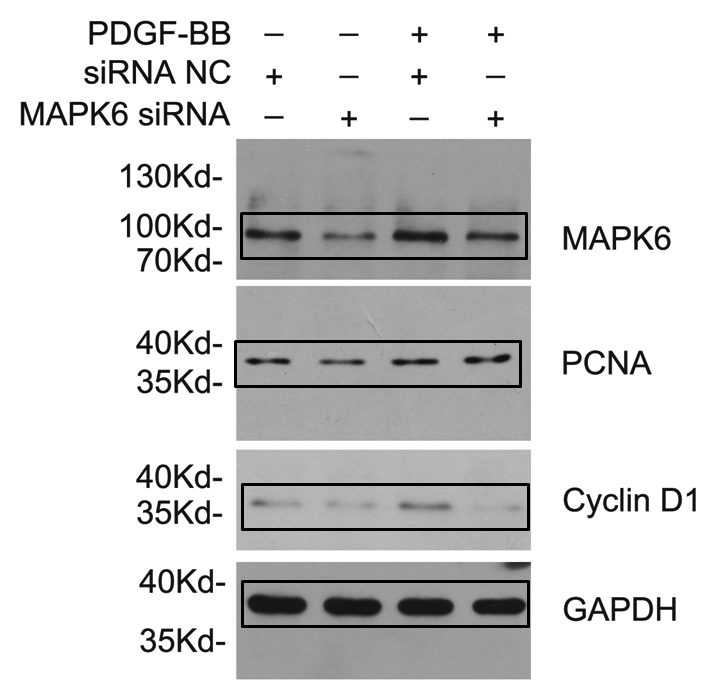


Supplementary Figure 2. Relative MAPK6, PCNA and CyclinD1 protein expressions analyzed with western blots in siRNA NC and MAPK6 siRNA with or without PDGF-BB lysates. GAPDH is the loading control. The boxed regions showed the content appearing in the figures in the main text.


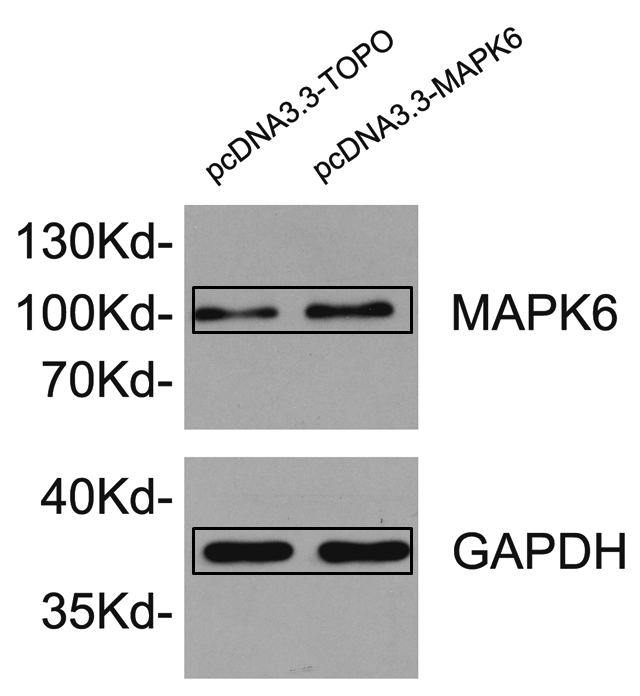


Supplementary Figure 3. Relative MAPK6 protein expressions analyzed with western blots in pcDNA3.3-TOPO and pcDNA3.3-MAPK6 lysates. GAPDH is the loading control. The boxed regions showed the content appearing in the figures in the main text.


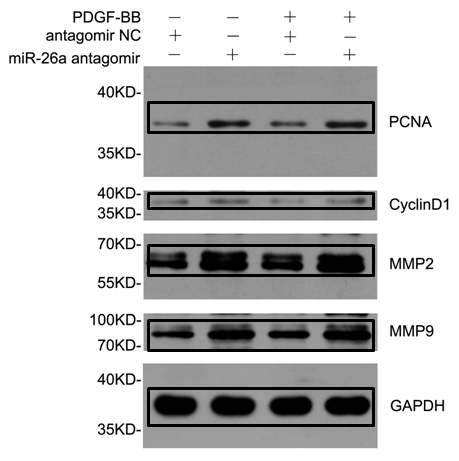


Supplementary Figure 4. Relative PCNA , CyclinD1, MMP2 and MMP9 protein expressions analyzed with western blots in antagomir NC and miR-26a antagomir with or without PDGF-BB lysates. GAPDH is the loading control. The boxed regions showed the content appearing in the figures in the main text.


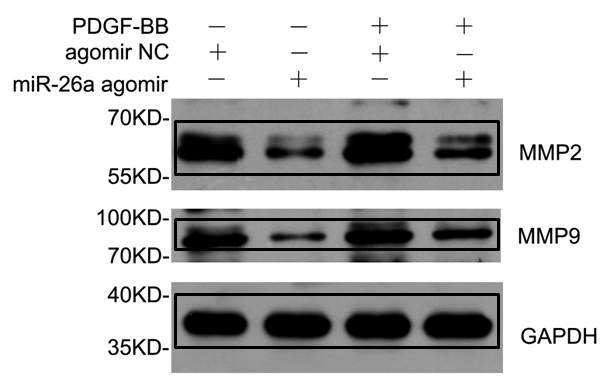


Supplementary Figure 5. Relative MMP2 and MMP9 protein expressions analyzed with western blots in agomir NC and miR-26a agomir with or without PDGF-BB lysates. GAPDH is the loading control. The boxed regions showed the content appearing in the figures in the main text.


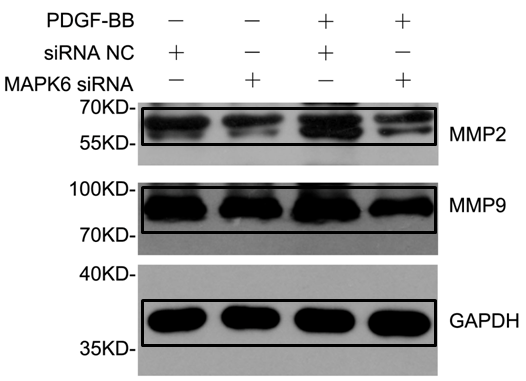


Supplementary Figure 6. Relative MMP2 and MMP9 protein expressions analyzed with western blots in siRNA NC and MAPK6 siRNA with or without PDGF-BB lysates. GAPDH is the loading control. The boxed regions showed the content appearing in the figures in the main text.
